# The future of Ukrainian healthcare: the digital opportunity

**DOI:** 10.7189/jogh.15.03039

**Published:** 2025-10-03

**Authors:** Gilbert H Mudge, Andrii Vilenskyi, Udai Kumar, Manish Kohli

**Affiliations:** 1 Harvard Medical School, Boston, Massachusetts, USA; 2 Superhumans Center, Lviv, Ukraine; 3 OHUM Healthcare Solutions, Maharashtra, India; 4 Pul Alliance for Digital Health and Equity and Fellow, Washington, D.C., USA

## Abstract

Following the dissolution of the Soviet Union in 1991, Ukraine inherited a decaying healthcare system. Significant reform was under way by 2014, laying the framework for creating a robust digital healthcare system. This viewpoint describes the evolution of this digital system and the constraints imposed by the ongoing war. Ukraine’s healthcare system is in a unique position to maximise its expsertise in digital healthcare, as it addresses workforce challenges and reconstructs its facilities.

The Russian Federation invaded Ukraine on 24 February 2022. The hallmark of the ongoing war has been its attack on civilians and civilian infrastructure, including brutal assaults on healthcare facilities and medical personnel. Conservative estimates from the first 11 months indicate that there were 707 assaults on healthcare facilities and 86 direct attacks on medical personnel, resulting in 62 deaths and 52 injuries [1]. Hospitals and clinics have been destroyed or shuttered, pharmacies closed, and medical supplies depleted. Medical care for military and civilian populations has been provided in basement facilities by a dwindling workforce, while medical care on the battlefield has been a source of inspiration for many [2].

As the war enters its fourth year, hope remains for both its end and the preservation of an independent Ukraine. As Ukraine’s healthcare system is eventually rebuilt and its workforce revitalised, a robust future-facing digital system will be essential. Ukraine’s prior expertise in digital healthcare will be central to these future efforts.

## HISTORY OF UKRAINIAN HEALTHCARE

Ukraine inherited a declining Soviet Union healthcare system after becoming independent in 1991. Despite aspirations for universal, free healthcare, the system remained underfunded and bureaucratic, with special interests and favouritism, and ‛care for cash’ being its defining features [3]. Political connections, rather than clinical need, governed the distribution of healthcare financial resources to public hospitals. Moreover, an average Ukrainian paid excessive prices for substandard care, and financial collapse was common. By 2014, a reform was under way at an astonishing pace [4]. Healthcare financing was redesigned, and by 2017, a single-payer National Health Service of Ukraine (NHSU) was initiated. Basic health services were to be available to all Ukrainians, and the government mandated that the electronic healthcare system serve as the sole platform for interaction between the NHSU and all medical service providers. Unfortunately, the Russian invasion abruptly halted this digital transformation.

The war has had a massive impact on the character of Ukrainian healthcare, but has not destroyed it entirely [4,5]. Medical doctors operate in basements and bomb shelters around the clock, adopting inventive approaches and providing complex, specialised care. New healthcare initiatives have also been launched [6], and rehabilitation facilities are being actively developed to deliver the best technology to wounded civilians and military personnel. Much of the medical workforce had to be transferred outside of the areas of conflict, while many of the female medical workers with children had evacuated the country early in the war, fleeing to western Ukraine or Europe.

The most pressing issues during wartime are the mental health of an entire nation, care of chronic diseases, cost escalation and availability of medicines and pharmaceuticals, and non-existent healthcare in the occupied territories. The World Health Organization (WHO) has prioritised the recovery of the Ukrainian healthcare system along four lines: service delivery, capital investment, health financing and strengthening institutions [7]. The more recent WHO reports detail the complexities of digital health [8–10].

## EVOLUTION OF THE CURRENT UKRAINIAN DIGITAL HEALTHCARE SYSTEM

In 2017, the Ukrainian Ministry of Health initiated the electronic healthcare system (EHS) e-Health project as a top priority [6]. The purpose was to maximise information exchange while reducing regulatory burdens of the older system. Until 2016, most public healthcare institutions did not have information technology, some institutions lacked personal computers, and internet access was irregular. Managers of health facilities lacked patient census to plan funding, and statistical data was received too late for quality management.

**Figure 1:**
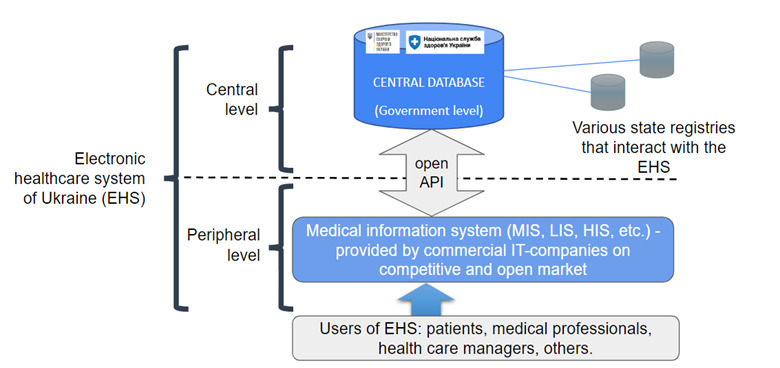
Two-level architecture of the electronic healthcare system of Ukraine. The electronic health system of Ukraine was divided into a central database and peripheral medical information systems developed by local IT companies. This allowed local needs to be addressed while establishing uniform national data acquisition. API – application programming interface, EMR – electronic medical record, MIS – medical information system.

The 2017 vision included a pan-Ukraine healthcare system with a registry of doctors, patients, and healthcare institutions to support national data collection. This included a central, national data repository and additional medical information systems (MIS) set up to address local needs. The latter were designed by IT companies on the open market, but were overseen by the government. By 2022, the central component had 36 million patients registered, with more than 1.6 billion electronic medical records and 400 000 active users. The EHS processed 1000–1500 requests on average to the central database per second (**Figure 1**). Patients have embraced digital healthcare services, as immediate benefits included planning an appointment, searching for drugs, creating an electronic prescription, and generating a sick list. This central structure is linked to over 40 regional MIS, which provide direct patient care and address local needs. The two-level electronic healthcare system has many advantages. The MIS transfer the same data in the same format to the central database; such a technical solution ensures the interoperability of all users. Moreover, local MIS can create unique functions and services, and managers of healthcare institutions and doctors direct them for the benefit of all patients.

## IMPACT OF WAR ON THE DIGITAL HEALTHCARE SYSTEM

Despite the war, the current system continues to have a broad impact on Ukrainian national healthcare. The EHS contains key healthcare registries of identified patients, healthcare facilities, managers, declarations, health professionals and specialists, prescriptions and electronic medical records. The created database allows for the automation of healthcare institutions and generates services for all patients. Such interactions are coordinated with other government registers; for example, the demographic register accounts for deceased patient numbers in prospective financial planning. During the COVID-19 pandemic, testing results and vaccination data were recorded centrally.

However, the IT companies, and hence the MIS, suffered huge financial losses, the destruction of infrastructure, and the evacuation of qualified IT specialists. The immediate impact has been to decrease the viability of IT companies and the number of MISs. Of the 40 MISs before the war, only four to six will probably survive. This is problematic for the future, and medical staff trained to work in one MIS will have to restructure work processes to another system.

Currently, the immediate goals include digital inventory management (e-Stock), modernisation of the state register of medicines, the state registry of medical equipment and devices, medical certificates for drivers, a rehabilitation initiative, blood donation management, a big data analysis platform, and the state registry of medical professionals. Expanding the mission beyond this will require peace.

## THE FUTURE CHALLENGES OF DIGITAL HEALTHCARE IN UKRAINE

There are inevitable challenges to the evolution of digital healthcare in Ukraine. The established digitalisation momentum may help embed digital health into national culture, but critical questions remain. What special efforts are needed to strengthen cybersecurity? Can these platforms be used to expand workforce innovation? Will there be national acceptance of skill shifting to accommodate workforce needs? Can the multiple MIS systems be simplified to one common pathway? Simply put, how can tragedy and adversity in Ukraine be transformed into an opportunity for digital innovation?

Looking ahead, what opportunities will digital health offer when resources for Ukraine’s reconstruction are so limited? Investing in modern medical and health information technologies unlocks an extraordinary potential, such as ensuring access to quality care for individuals, regardless of their location or circumstances. A robust digital infrastructure is the cornerstone of transforming fragmented healthcare ecosystems into cohesive, efficient, and value-maximising care continuums. Digital health innovations are not merely tools and Ukraine’s digital opportunity; they are catalysts for profound change, shifting the paradigm from traditional, facility-based care to patient-centred solutions that deliver services directly at the source. This is the digital opportunity for Ukraine.

## CONCLUSIONS

The Ukrainian healthcare system currently faces formidable challenges to care for the civilian population and military wounded. However, it also faces challenges of rebuilding, yet bricks, mortar, and workforce redesign will not solve the current devastation. The six-year history of digital system reflects vision and innovation that should allow it to circumvent the organisational structure and dysfunction of so many established healthcare systems. Ukraine’s digital healthcare legacy should be part of its healthcare reconstruction.
